# Injection Volume Is a Better Predictor of Stiffness Restoration Than Injection Force in an In Vitro Study of Nucleus Augmentation of the Intervertebral Disc

**DOI:** 10.1002/jsp2.70081

**Published:** 2025-06-10

**Authors:** J. P. Warren, A. R. Dixon, M. P. Culbert, A. Khan, M. Mengoni, R. K. Wilcox

**Affiliations:** ^1^ University of Leeds, Institute of Medical and Biological Engineering Leeds UK; ^2^ Leeds Centre for Neurosciences, Leeds Teaching Hospitals NHS Trust Leeds UK

**Keywords:** biomaterials, biomechanics, intervertebral disc degeneration, nucleus augmentation, surgical optimisation

## Abstract

**Purpose:**

Nucleus augmentation has been proposed as an early‐stage intervention for intervertebral disc degeneration and involves the injection of a biomaterial into the nucleus to restore disc height and functionality. The aim of this work was to identify clinically relevant quantitative measures that indicate the mechanical performance of the disc following nucleus augmentation.

**Method:**

Bovine tail bone‐disc‐bone units (*n* = 22) were mechanically tested under cyclic loading sequentially in native, artificially degenerated, and treated states. Treatment involved injection of a peptide‐glycosaminoglycan mixture into the degenerated disc to a predetermined load using a syringe driver with an integrated force sensor. The stiffness restoration of the treatment was determined by comparing the biomechanical behavior of the native state to the treated state of each disc. The stiffness restoration was then compared against clinically quantifiable parameters.

**Results:**

No significant biomechanical differences were observed between the native and treated states, but both were significantly different from the degenerated state. The force delivered during injection was found to ramp to a steady state, followed by a final rapid increase; however, all measures associated with injection force poorly correlated with the level of stiffness restoration. Volume injected and change in disc height from injection had the strongest relationship to stiffness restoration.

**Conclusion:**

This work showed that measuring the injection force for injectable treatments of the disc can provide lower and upper limits for delivery, but direct measures are stronger indicators of disc stiffness restoration.

## Graphical abstract

This study investigated nucleus augmentation as an early intervention for intervertebral disc degeneration by injecting a biomaterial to restore disc height and functionality. Mechanical tests on bovine tail discs showed that treated discs exhibited similar stiffness to native discs, differing significantly from degenerated ones. The volume injected and change in disc height were the strongest indicators of stiffness restoration, while injection force had poor correlation with stiffness outcomes.

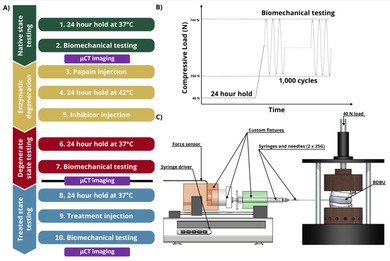

## 1 Introduction

Nucleus augmentation, in which a biomaterial is injected into the intervertebral disc, has been proposed as an early stage intervention for disc degeneration [1]. A number of injectable biomaterials are under development for nucleus augmentation, and studies have reported on both their biological and mechanical characteristics [2, 3]. The biomechanical performance of intervertebral disc specimens augmented with candidate biomaterials has also been evaluated through a range of in vitro tests in cadaveric human and animal tissue, with some evidence of the ability of the treatment to restore short‐term biomechanical function [4]. In these studies, the amount of biomaterial injected in the nucleus augmentation procedure has been either to a fixed volume or based on the haptic judgment of the researcher [2], and there has been little investigation to date on the effects of clinical variables such as the volume of injected material or the force required to deliver it.

In a clinical setting, if the volume of biomaterial injected into the nucleus were too low, then there would be less restoration of disc height or biomechanical function, while too great a volume could potentially increase the risk of herniation or end‐plate fracture [5]. However, the optimum volume to inject would likely be governed by the size, extracellular constituents, and degenerative state of the disc. An alternative may be to measure the mechanical resistance of the disc to filling by monitoring the force required to deliver the biomaterial [6].

The aim of this study was to investigate the relationship between clinically quantifiable measures of biomaterial delivery in nucleus augmentation and the resulting mechanical performance of the augmented intervertebral disc. The study was conducted using a bovine in vitro pre‐clinical biomechanical model to allow the greatest control of confounding factors over a short testing duration and a previously developed peptide‐glycosaminoglycan (GAG) injectable hydrogel [7, 8].

## 2 Materials and Methods

### 2.1 Specimen Preparation

Bovine tails (sourced from a local abattoir—Penny & Sons, UK) (C1‐C4) (*n* = 12) were imaged under microcomputed tomography (μCT), and sectioned into bone‐disc‐bone units (BDBUs) retaining a consistent thickness of vertebral bone [9]. The exposed bone was cleaned using a surgical water pik (Pulsavac Plus Wound Debridement System, Zimmer Biomet, USA) and the specimens were placed in an agitated anticoagulant bath at 4°C for 24 h (sodium citrate, 20.5 mM). Specimens were rinsed and frozen to −80°C until the day of testing.

### 2.2 Overview

**FIGURE 1:**
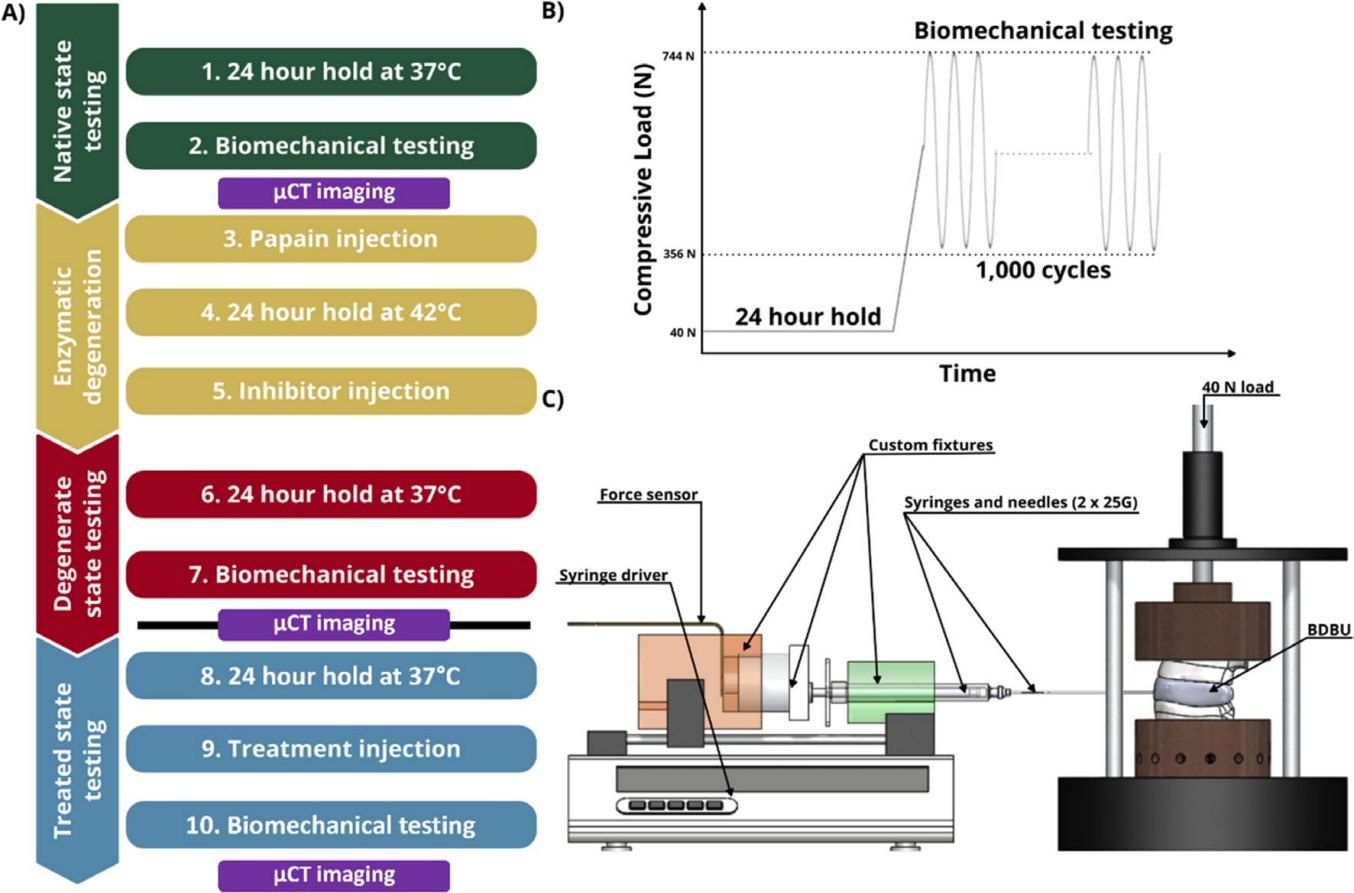
A) Summary flow chart of test procedure, black line shows point where specimens are frozen then thawed to continue process B) hold period and biomechanical test compressive loading, and C) treatment injection fixture set up (not to scale).

The prepared BDBUs were subjected to a sequential testing protocol, where the units were biomechanically tested in the native state, subjected to a rapid enzymatic degeneration procedure, tested in the artificially degenerated state, injected with a hydrogel, and finally tested in the treated state. A summary of the testing is shown in Figure 1A. The testing protocol was carried out continuously from Steps 1 to 7; then a freeze/thaw cycle was carried out before the protocol was continued from Step 8 to the end.

### 2.3 Biomechanical Testing and Imaging

Since each BDBU was biomechanically tested in the native, degenerate, and treated states, it was important to ensure consistency of hydration of the intervertebral disc tissue between tests. The mechanical testing protocol was described in [10], with key aspects summarized here. As part of the protocol, the BDBUs were not potted to ensure maximum fluid flow through the tissue to prevent dehydration during testing. To establish a physiologically relevant osmotic equilibrium, the BDBUs were held for a 24 h period in a phosphate buffer saline (PBS) bath under a 40 N load in a holding rig prior to each testing phase (steps 1, 6, and 8 in Figure 1A). This load corresponded to an intradiscal pressure (IDP) of approximately 0.1 MPa for an average sized bovine tail disc [11], which represented lying in a supine position [11, 12, 13, 14]. After the hold period, the BDBUs were transferred to an electromechanical testing machine (ElectroPuls E10000, 2527 Series 10 kN Bi‐axial Dynacell, Instron, USA). The specimens were positioned in a PBS bath between bespoke manufactured ventilated stainless steel fixtures to allow fluid flow through the exposed bone surfaces; the PBS bath was held at 37°C throughout testing. Each specimen was then subjected to a 1000‐cycle cyclic compression test at 1 Hz between 356 N and 744 N as shown in Figure 1B (steps 2, 7, and 10). The upper limit corresponded to an estimated IDP of 2.3 MPa, which represented carrying 20 kg of weight. The lower limit corresponded to an estimated IDP of 1.1 MPa, which represented relaxed standing [12, 13, 14]. A test duration of 1000 cycles has been used previously for similar biomechanical testing of spinal units [4, 15, 16, 17, 18]. After each cyclic compression test, specimens were wrapped in PBS soaked paper tissue to maintain hydration and then imaged using μCT (74 μm cubic voxel size, 114 μA, 70 kV; μCT100, Scanco Medical AG, Switzerland). From the μCT images, the disc height was measured as the smallest distance between the two endplates (EPs) in the mid‐sagittal and mid‐coronal planes. This was usually close to the center of the disc in both planes.

### 2.4 Enzymatic Degeneration

After the native state testing, the BDBUs were immediately artificially degenerated by injecting 0.3 mL papain (1.6 kU/ml) with a 30G needle to non‐selectively break down the collagen and proteoglycan structures within the nucleus [19]. The units were then left to digest for a 24 h hold period under 40 N load at 42°C (step 4) and then 0.3 mL of a papain inhibitor, ebselen (0.064 μmol), was injected, preventing further degeneration and left for a 24 h hold period under 40 N load at 37°C (step 5). Following biomechanical testing (steps 6 and 7), the units were frozen to −80°C until ready for treatment.

### 2.5 Nucleus Augmentation

Specimens were thawed and then left for a 24 h hold period under 40 N load at 37°C (step 8). The nucleus augmentation procedure was carried out between the hold period and the cyclic compression test (Step 9). The augmentation was performed using a peptide‐GAG hybrid hydrogel (P_11_‐12 and chondroitin sulfate, 1:20 ratio [7, 8]). Lyophilised peptide (P_11_‐12, Ac‐SSRFOWOFESS‐NH_2_) was weighed out (20 mg, 0.014 mol, 95% purity, CS Bio, CHEMGO Organica AG, Switzerland) into glass vials and reconstituted in 130 mM NaCl aqueous solution (500 μL). Lyophilised chondroitin sulfate (CS) (MW ~ 50 kDa, ZPD, Zeria Pharmaceutical Co. Ltd., Denmark) was weighed out (137 mg) into glass vials and reconstituted in 130 mM NaCl aqueous solution (500 μL). The samples were then vortexed (30s) at 2500 rpm on a vortex mixer. After early analysis of the augmented discs, an additional step was added to sonicate the peptide and GAG components to reduce the likelihood of microbubble formation. The peptide and GAG components were injected separately via two 25G needles into the centre of the nucleus pulposus, such that mixing and gelation occurred in situ. To allow radiographic analysis of the injected material in the post‐test μCT scans, a radio‐opaque agent (Ultravist) was added to the gel components (50% dilution in 130 mM NaCl aqueous solution from stock solution—38.5% w/v, 185 mg I/ml, 500 μL). The injection was performed using a custom rig that connected two syringes in parallel to a syringe driver such that both components were delivered at a constant rate. A 40 N axial compressive load was applied to the BDBU during injection using calibrated weights attached to a bespoke manufactured rig, as shown in Figure 1C. A transducer (Flexiforce B201 series, Tekscan Inc., USA) was inserted between the syringe driver and the rear of the custom injection rig to measure the force applied to the syringes (‘injection force’). The volume of hydrogel injected was measured using the syringe markings. Injection was stopped when a load of 80 N was reached [20], the syringes were empty, or if the syringe driver began to slip repeatedly. The pre‐ and post‐injection BDBU height were recorded using Vernier calipers.

### 2.6 Data Analysis

Specimens were excluded from the analysis if they presented less than 10% change in stiffness following the degeneration procedure, or if the post‐test radiographic analysis revealed that either the injections were not into the nucleus or there were observable coalesced air microbubbles.

A total of 22 specimens were analyzed. Using an ad‐hoc script (MATLAB and Statistics Toolbox Release 2021a, The MathWorks Inc., Natick, Massachusetts, United States), the stiffness of each individual loading cycle was computed from a linear fit of the load–displacement data, excluding 5 points of the extreme values (corresponding to approximately 10% of the cycle at the beginning and the end of the loading phase). From each 1000 cycles of testing for each BDBU state, the mean stiffness over the last ten cycles was extracted for comparison between samples and states (hereafter referred to simply as “stiffness”). Stiffness restoration following treatment was defined as the difference between the native and treated stiffness.

Due to the paired and non‐normal nature of the data, a Friedman test was used to compare the different states (native, degenerate and treated), with post hoc Wilcoxon signed‐rank tests (*α* = 0.05). All specimens were placed into two sets of groupings (for which the limits were chosen to have even‐sized groups) related to the total volume of hydrogel injected (low < 0.85 mL, *n* = 7; 0.85 mL< medium < 1.25 mL, *n* = 7; high > 1.25 mL, *n* = 8) and to the maximum injection force achieved recorded during the delivery of the hydrogel to each degenerate sample as a treatment (low < 30 N, *n* = 7; 30 *N* < medium < 40 N, *n* = 7; high > 40 N, *n* = 8). Due to the small sample sizes in the subgroups and the nature of the data, only the median, maximum, and minimum values for these subgroups were investigated. Statistical analysis was performed using the Statistics Toolbox in MATLAB (v 2021a).

The stiffness restoration was evaluated against the six measurable or calculable parameters using linear regressions: volume injected, volume injected/disc cross‐sectional area, injection force, injection force/disc cross‐sectional area, work done (integral of injection force‐time plot), and recovery of BDBU height following injection (post‐injection height minus pre‐injection height).

All data in this study is available open access at the Leeds data repository [21].

## 3 Results

**FIGURE 2:**
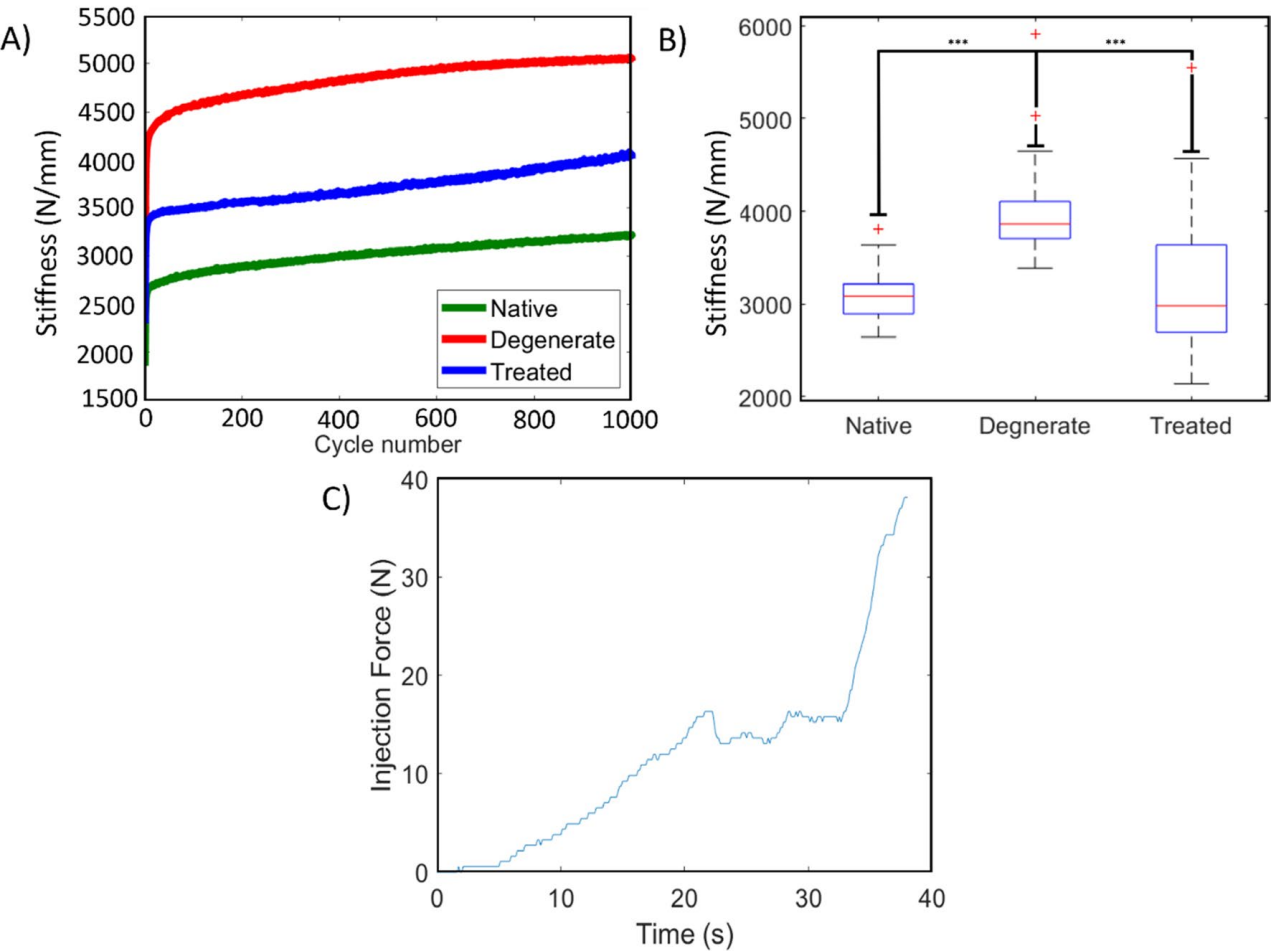
(A) Typical plot of the specimen stiffness over each of the 1000 loading cycles for the three different states. (B) Comparison of stiffness in different states for all specimen in box plot format. Red cross (+) indicates outliers in data analysis. Statistical significance between native—degenerated and degenerated—treated states, *** = *p* < 0.001. (C) Typical plot of the force applied to the syringe during the injection.

**FIGURE 3:**
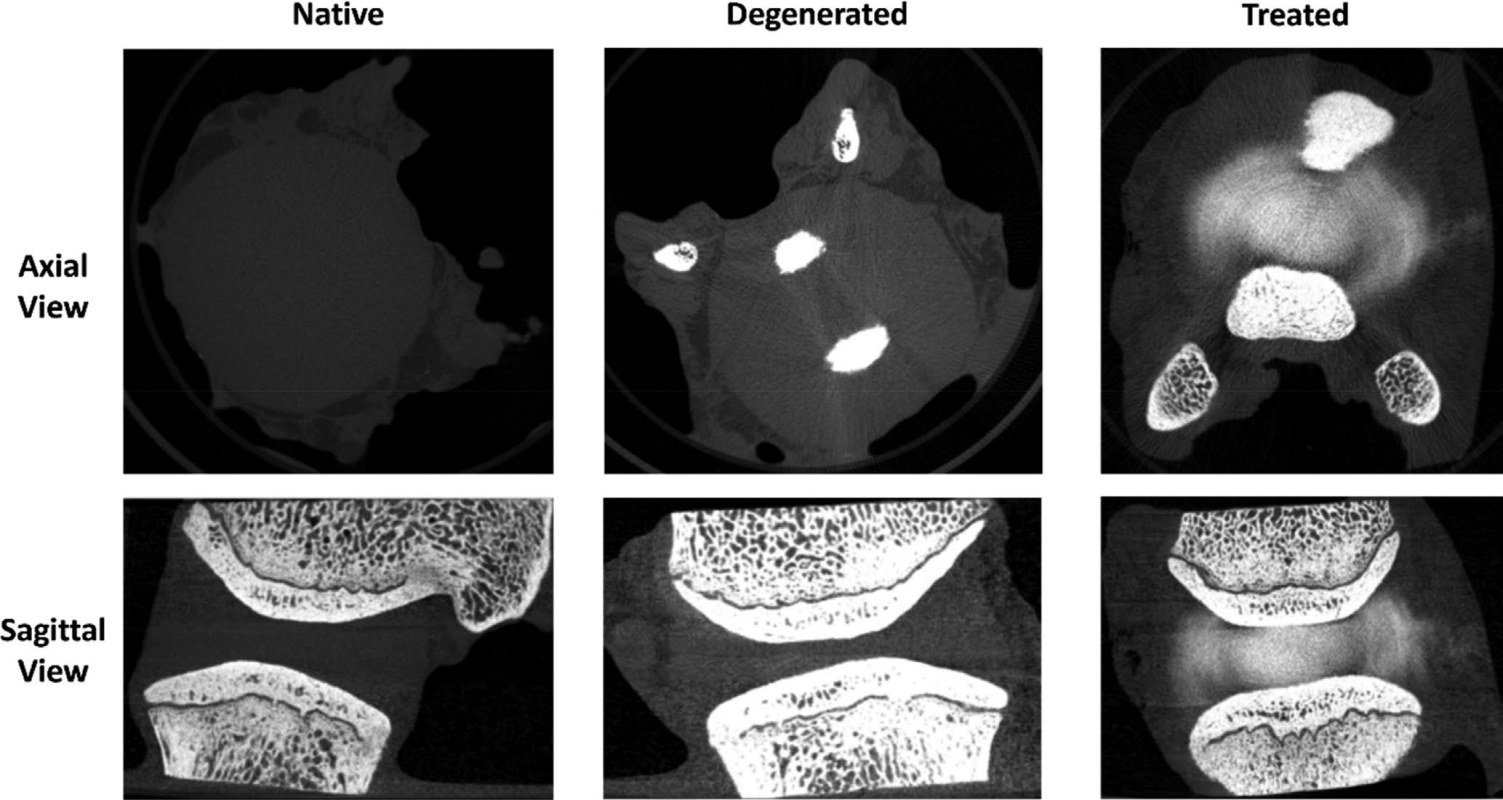
Representative μCT micrographs of a bone‐disc‐bone specimen in a native, degenerated and treated state. The axial and sagittal views of the specimen show the location and distribution of the hydrogel post‐injection to the nucleus and inner annulus region.

In all specimens and states, the stiffness increased throughout the duration of the cyclic loading. The stiffness in the degenerated state was higher than in the native state for each specimen (Figure 2A). Significant differences were found (Figure 2B) in stiffness values between the degenerate and the native states (post hoc Wilcoxon test, *p* < 0.001) as well as between the degenerate and the treated states (post hoc Wilcoxon test, *p* < 0.001), while there was no significant difference between the treated and native states (post hoc Wilcoxon test, *p* = 0.78). During the nucleus augmentation procedure, there was an initial ramp in the injection force, followed by a period where force remained relatively steady before it rose more steeply (Figure 2C). The hydrogel was successfully injected into the nucleus of each specimen. From the μCT images, the location and distribution of the hydrogel were found to be consistent across all samples, even those with low injection volumes (Figure 3). The hydrogel was localized to the nucleus with some permeation into the inner annulus. No hydrogel was visible in the outer annulus of any specimen post‐test, indicating no visible expulsion occurred during cyclic testing.

**FIGURE 4:**
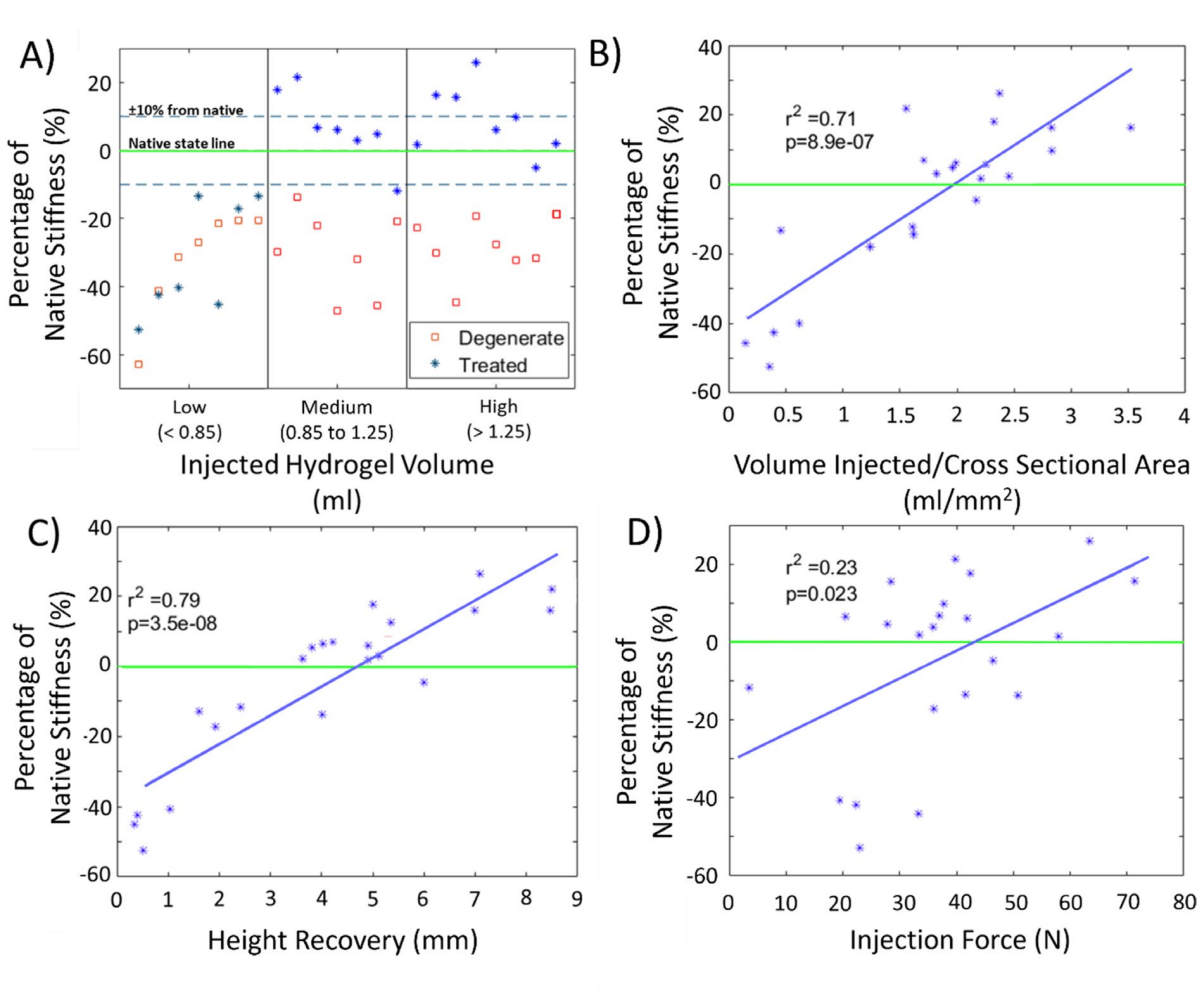
A) Summary of stiffness differences compared to native state for degenerate and low, medium, and high injected hydrogel volume specimens (note points are distributed uniformly on x‐axis for visualization), B) Linear regression relationship between percentage of native stiffness and volume/cross sectional of treated specimens, C) Linear regression relationship between percentage of native stiffness and post injected disc height recovery of treated specimens, D) Linear regression relationship between percentage of native stiffness and injection force.

The median (max, min) of the stiffness restoration for the low, medium, and high volume groups was (Figure 4A): −41.3 (−13.4, −52.7)%, 6.1 (21.6, −13.5)%, and 6.0 (25.7, −11.8)% respectively. Similarly, the low, medium, and high injection force groups median (max, min) restoration values were: −11.8 (15.5, −52.7)%, 3.1 (21.6, −45.1)%, and 3.8 (25.7, −13.5)% respectively.

Stiffness restoration correlated well with the injected volume normalized by cross‐section area and with the disc height recovery. The correlation was good but weaker for the injected volume and was weak for maximum injection force or the work done. The correlations and significance values are summarized in Table 1.

**TABLE 1:** Correlation and statistical significance of correlations between stiffness restoration and various parameters.

Parameter	r^2^	*p*
Injected volume	0.63	1.20E‐05
Volume/cross‐section area	0.71	9.10E‐07
Disc height recovery	0.79	3.50E‐08
Maximum injection force	0.23	2.30E‐02
Work done	0.31	6.70E‐03

## 4 Discussion

The objective of this study was to explore how various measurable clinical factors relate to the mechanical performance of the intervertebral disc after nucleus augmentation. Three key variables were measured: the volume injected, the injection force, and the change in disc height following augmentation. The results showed that the nucleus augmentation procedure was able to provide mechanical restoration by reducing the disc stiffness from the degenerate state to that of the native state, with no statistically significant difference found across all the data between the native and treated states. When divided into the subsets based on the volume injected, it was found that a minimum volume was required to achieve stiffness restoration (Figure 4A).

The artificial degeneration model used had the advantage of enabling the same specimens to be tested sequentially in different states and is similar to other work where either enzymatic degeneration (protease‐based [22, 23, 24, 25, 26, 27, 28] or chondroitinase‐based [29, 30]) has been used. However, the limitation of the approach is that the degeneration was localized within the nucleus due to the injection of the enzyme and does not represent degenerative changes in the annulus such as tears and fissures that are observed clinically [31, 32]. The model is therefore representative of early degeneration with minimal annular pathology [33] which would be a likely target for nucleus augmentation treatment.

Previous in vitro studies of nucleus augmentation have stopped injection based either on reaching a given volume [7, 22, 25, 28, 30, 34, 35, 36] or on haptic feedback [18, 24, 26, 27, 29, 37, 38, 39, 40, 41], but there is a lack of consistency in how these measures have been reported. The current work uniquely assessed both the volume and the force of injection, using these measures as variables of interest for examining the biomechanical restoration. Reporting both the injection volume and injection force allowed for preliminary conclusions to be drawn about which variable has the potential as the better clinical factor. For the biomaterial used in this work, the relationship between the direct volume and height measurements and the level of stiffness restoration was stronger than for the measurements derived from the applied injection force. Clinically, the volume of injected material can be monitored directly on syringes and change in disc height may be approximated with fluoroscopy or other available imaging modalities during surgery. In this study, the change in disc height was calculated in the centre of the disc where the endplates are closest because this distance could be measured consistently. Due to the curved nature of the endplates in the bovine model and variances in the amount of curvature between specimens, it was not possible to derive a consistent ‘average’ disc height measurement across the cross‐section. Similarly, although the cross‐sectional area could be measured from the μCT at the midplane of the disc, it was difficult to identify the peripheral annulus regions near the endplates and so the disc volume was also not calculated. Consequently, only the disc cross‐sectional area rather than the disc volume was used to normalize the injection volume, and the stiffness was not normalized (i.e., as an apparent modulus) to the disc dimensions. Clinically, there could be potential to better estimate all of the disc dimensions from additional magnetic resonance imaging [42].

The injection force was monitored to provide a quantitative measure of haptic feedback. It was found that, as the hydrogel was injected, the injection force initially stabilized before rising steeply. However, the maximum force did not correlate well with biomechanical function restoration in this in vitro model. It is likely that in vivo, the progressive degeneration of the disc and remodeling of the surrounding tissues would provide greater resistance to injection in the initial stages, and the injection force would be more difficult to interpret. Further, the injection force would depend on intervertebral disc (IVD) pressure, which varies between patients and positions [12, 13, 14]. Thus, injection force alone may not provide sufficient data to be a suitable real‐time clinical measurement tool. Nevertheless, when used in conjunction with the directly measured volume and height parameters, the injection force could still provide a meaningful upper limit measurement to prevent damage.

In this study, the biomaterial used for the nucleus augmentation was a previously developed peptide‐GAG hybrid hydrogel [7, 8]. The GAG component acts both to trigger rapid self‐assembly of the peptide and also to mimic the healthy nucleus tissue's ability to imbibe water. By having a similar GAG composition to the native nucleus and a hydrogel with a high water content, the aim was that the natural balance between the load and osmotic pressure would reduce risks associated with overfilling. It should, therefore, be noted that while the results of this study are applicable to other nucleus augmentation materials, the ranges of the measured injection parameters likely depend on the biomaterial used and its properties. Materials with different osmotic potential, gelation mechanisms, or mechanical properties could result in different biomechanical outcomes for a given injected volume. The needle gauge and length used for injection will also affect the applied forces. Although the relationships between injection parameters and resultant biomechanical performance may be unique to a given material, the parameters and techniques developed in this work could readily be applied to other biomaterials.

## 5 Conclusions

This study clearly demonstrates that mechanical restorative outcomes for nucleus augmentation vary with injection parameters. Specifically, in this in vitro bovine model, the volume injected and change in IVD height were found to be suitable predictors of biomechanical outcome, while injection force may provide an additional safety indication to prevent damage during a nucleus augmentation.

## Author Contributions

Conceptualization (**A.R.D., J.P.W., M.M., R.K.W**.); Data curation (**A.R.D., R.K.W**.); Formal Analysis (**A.R.D., J.P.W., M.M., R.K.W**.); Funding acquisition (**R.K.W**.); Investigation (**A.R.D., J.P.W., M.P.C**.); Methodology (**A.R.D., J.P.W., M.P.C**.); Project administration (**R.K.W**.); Supervision (**M.M., R.K.W**.); Visualization (The authors have nothing to report); Writing – original draft (**A.R.D., J.P.W**.); Writing – review and editing (**A.R.D., J.P.W., M.P.C., A.K., M.M., R.K.W**). **A. Dixon** and **J. P. Warren** contributed equally to this paper. For the purpose of open access, the authors have applied a Creative Commons Attribution (CC BY) license to any Author Accepted Manuscript version arising from this submission.

## Conflicts of Interest

The authors declare no conflicts of interest.

## Data Availability

The data associated with this paper are openly available from the University of Leeds Data Repository at https://doi.org/10.5518/1712 [21].
